# The effect of offset cues on saccade programming and covert attention

**DOI:** 10.1177/1747021818759468

**Published:** 2018-03-01

**Authors:** Daniel T Smith, Soazig Casteau

**Affiliations:** Department of Psychology, Durham University, Stockton-on-Tees, UK

**Keywords:** Saccade, oculomotor, eye-movement, attention, cueing, priming

## Abstract

Salient peripheral events trigger fast, “exogenous” covert orienting. The influential premotor theory of attention argues that covert orienting of attention depends upon planned but unexecuted eye-movements. One problem with this theory is that salient peripheral events, such as offsets, appear to summon attention when used to measure covert attention (e.g., the Posner cueing task) but appear not to elicit oculomotor preparation in tasks that require overt orienting (e.g., the remote distractor paradigm). Here, we examined the effects of peripheral offsets on covert attention and saccade preparation. Experiment 1 suggested that transient offsets summoned attention in a manual detection task without triggering motor preparation planning in a saccadic localisation task, although there were a high proportion of saccadic capture errors on “no-target” trials, where a cue was presented but no target appeared. In Experiment 2, “no-target” trials were removed. Here, transient offsets produced both attentional facilitation and faster saccadic responses on valid cue trials. A third experiment showed that the permanent disappearance of an object also elicited attentional facilitation and faster saccadic reaction times. These experiments demonstrate that offsets trigger both saccade programming and covert attentional orienting, consistent with the idea that exogenous, covert orienting is tightly coupled with oculomotor activation. The finding that no-go trials attenuates oculomotor priming effects offers a way to reconcile the current findings with previous claims of a dissociation between covert attention and oculomotor control in paradigms that utilise a high proportion of catch trials.

## Introduction

Humans exist in a complex visual environment. Given the limitations on information processing capacity, a key challenge faced by the visual system is the selection of task-relevant visual signals from irrelevant noise. One way to achieve this selection is to orient attention to the location of the relevant signal. Orienting of attention can be driven endogenously, in response to our current goals (e.g., looking up and down a street before crossing) or exogenously, in response to a salient event in the environment (e.g., orienting to a flashing light in the rear-view mirror) (Posner & Cohen, 1980). Both modes of orienting can occur overtly, by moving the eyes to fixate the relevant location. However, orienting can also be covert, such that the “spotlight” of attention is moved while the eyes remain fixated.

Although covert attentional orienting occurs in the absence of overt eye-movements, covert and overt orienting share some common processes (Awh, Armstrong, & Moore, 2006; Smith & Schenk, 2012). Indeed, one widely held view is that covert attentional orienting depends on the activation of the oculomotor system (Klein, 1980; Sheliga, Riggio, & Rizzolatti, 1994). This strong view of the coupling between attention and eye-movements is controversial and a number of authors have argued that endogenous covert attention can be deployed in the absence of motor activation. For example, Klein and colleagues reported that covertly attending a peripheral location did not facilitate saccadic reaction times (SRTs), which it should do, if covert attention is the same as motor preparation (Hunt & Kingstone, 2003; Klein, 1980; Klein & Pontefract, 1994; MacLean, Klein, & Hilchey, 2015). Similarly, Belopolsky and Theeuwes (2012) have shown that maintenance of attention is independent of saccade programming; Born, Mottet, and Kerzel (2014) have demonstrated that motor preparation was not sufficient to orient attention; and Dunne, Ellison, and Smith (2015) reported that instrumental conditioning of eye-movements modulated saccade latencies but not covert orienting of attention. In related work, we demonstrated that disrupting saccade preparation by presenting stimuli beyond the range of saccadic eye-movements interferes with exogenous orienting to peripheral onsets but not endogenous orienting to symbolic cues (Smith, Rorden, & Schenk, 2012) or gaze cues (Morgan, Ball, & Smith, 2014). The same manipulation affects exogenous orienting in feature search but not endogenous orienting in conjunction search (Smith, Ball, & Ellison, 2014; Smith, Ball, Ellison, & Schenk, 2010) and encoding and rehearsal of spatial, but not visual working memories (Ball, Pearson, & Smith, 2013; Pearson, Ball, & Smith, 2014). This pattern of specific disruption to exogenous attention by disruption to the oculomotor system can also be observed in clinical populations; patients with oculomotor deficits typically present with defective exogenous orienting but largely preserved endogenous orienting (Gabay, Henik, & Gradstein, 2010; Rafal, Posner, Friedman, Inhoff, & Bernstein, 1988; Smith, Rorden, & Jackson, 2004), although see Craighero, Carta, and Fadiga (2001). These studies have led to the proposal that exogenous attention is tightly coupled to the oculomotor system, whereas endogenous orienting is largely independent of oculomotor control (Smith & Schenk, 2012).

One problem with the conclusion that exogenous orienting is causally linked to motor preparation comes from the observation that some types of cue can elicit exogenous orienting seemingly without activating a saccade plan. For example, peripheral offsets reliably summon covert attention in a Posner-style cueing task (Hopfinger & Mangun, 1998, 2001; Pratt & McAuliffe, 2001; Riggio, Bello, & Umilta, 1998) but do not reliably generate a remote distractor effect (RDE) (Hermens & Walker, 2010; Todd & Vangelder, 1979), unless the stimuli are defined by contrast rather than colour (Ludwig, Ranson, & Gilchrist, 2008). Furthermore, the cost of making antisaccades is significantly reduced if the saccade endpoints are indicated by object offset rather than object onset, suggesting that onsets exert a much more powerful influence on saccade programming than offsets (Pratt & Trottier, 2005). Studies using visual search also indicate that an object offset is less likely to elicit saccadic programming than an object onset. For example, object disappearances do not elicit reflexive saccades in visual search (Boot, Kramer, & Peterson, 2005), unless the offset reveals another object (Brockmole & Henderson, 2005). Similarly, short-wavelength colour cues (s-cone stimuli) do not retard SRTs when used as a distractor in the remote distractor paradigm (RDE), leading some authors to conclude that they do not elicit activation in the structures critical for the computation of saccade parameters such as the Superior Colliculus. However, the same stimulus does elicit exogenous shifts of attention (Sumner, Adamjee, & Mollon, 2002). Together, these studies suggest that some classes of peripheral cues, such as offsets and s-cone stimuli, can reliably summon covert attention while only producing minimal activation of the oculomotor system.

The claim that offsets can reliably capture attention without reliably engaging the oculomotor system is potentially problematic for theories of attention that propose a mandatory coupling between the two processes (Klein, 1980; Rizzolatti, Riggio, & Sheliga, 1994; Smith & Schenk, 2012). However, to date no study has explicitly examined the effects of offset cues on exogenous attentional facilitation and saccade programming within the same study. Here, we address this question using the Posner cueing task. We operationalised attentional facilitation as faster and more accurate manual RTs in covert detection (Experiment 1) and discrimination (Experiments 1, 2, and 3) tasks, and saccade programming as faster and more accurate saccades in a saccadic localisation task. The claim that offsets can summon attention without triggering saccade programming leads to a clear prediction; there should be attentional facilitation in the manual detection and discrimination task but no facilitation of SRT in the saccadic localisation task.

## General method

### Participants

In total, 19 undergraduate volunteers (14 female, median age 19 years, 15 right handed) took part in Experiment 1 and 10 other volunteers (5 female, median age 25 years, 8 right handed) from Department of Psychology, Durham University took part in both Experiments 2 and 3. All participants had normal vision or wore contact lenses to correct their vision. All participants gave informed consent to participate. The study was approved by the Department of Psychology Research Ethics Committee and was conducted in accordance with the British Psychological Society (BPS) code of ethics.

### Apparatus

Stimuli were generated using a Cambridge Research Systems ViSaGe graphics card and displayed on a 17-inch Sony Trinitron cathode ray tube (CRT) monitor with a refresh rate of 100 Hz. Manual responses were collected using a two-button response box. Eye-movements were recorded using a Cambridge Research Systems Video Eyetracker Toolbox sampling at 250 Hz.

#### Stimuli and general procedure

The placeholders were black squares subtending 2° of visual angle. The fixation point was a 0.3° black spot surrounded by a black square subtending 2°. The peripheral cue was the disappearance of one of the two peripheral placeholders (Experiments 1 and 2) or the permanent offset of one of the peripheral placeholders (Experiment 3). The central cue was the disappearance of the box surrounding the fixation point. The target in the Saccadic Localisation and Manual Detection tasks was a light grey annulus (75 cd/m^2^, diameter 1.5°). In the Discrimination task, the target was a filled white bar (100 cd/m^2^, 0.5° x 1.5°). The background was grey (54 cd/m^2^). The viewing distances were 57 cm (Experiment 1) and 50 cm (Experiments 2 and 3).

The participant was seated on an adjustable chair in a dimly lighted room. After setting up the eye tracker, a 12-point calibration phase began. If the calibration was unsatisfactory, another calibration phase was initiated. Otherwise, a block of trials began. Blocks of trials for each condition were completed consecutively and the order in which the different conditions were presented was counterbalanced across participants.

**Figure 1.**
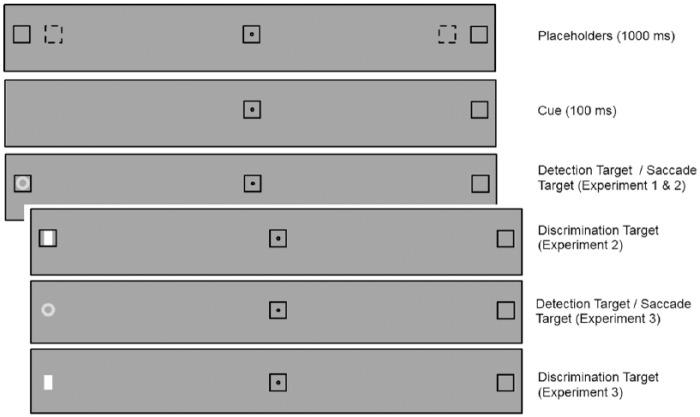
Schematic of a trial from the valid condition illustrating the timing and stimuli used in Experiments 1, 2, and 3. Only one stimulus eccentricity was used in Experiments 2 and 3. The dotted squares on the top panel indicate the 8° eccentricity condition.

Response types (Manual Detection [Experiments 1, 2, and 3], Manual Localisation [Experiments 2 and 3], Manual Discrimination [Experiments 2 and 3] or Saccade [Experiments 1, 2, and 3]) were tested in different blocks. Trials began with the onset on the fixation point and three placeholders. The centres of the peripheral placeholders were presented at an eccentricity of 8° (Experiments 1, 2, and 3) or 10° (Experiment 1) from fixation in left and right hemifields. After 1000 ms, one of the locations was cued (i.e., transient offset or permanent offset of placeholders) during 100 ms. The target was then presented simultaneously with the re-appearance of the placeholder (except for Experiment 3) and remained visible until a response was made. Figure 1 illustrates the sequence of events in a typical trial.

## Analysis

In Experiment 1, one participant withdrew after completing two blocks of trials and was excluded from the analysis and another participant had false alarm rates of >33% in the Manual Response condition and was also excluded.

In the Manual Response condition, trials were rejected when (a) blinks, loss of eye tracking or other artefacts made it impossible to determine whether a saccade had been executed, (b) participants broke fixation in manual condition, and (c) had a reaction time (RT) of <100 ms. This resulted in the exclusion of ~1% of trials in each of the three experiments. In the Saccade condition, trials were rejected when (a) blinks, loss of eye tracking or other artefacts made it impossible to determine whether a saccade had been executed (1.7% of trials in Experiment 1, 1.8% in Experiment 2%, and 10.5% in Experiment 3), (b) the saccade was made prior to target presentation (3.9% of trials in Experiment 1, 3.1% in Experiment 2%, and 2.8% in Experiment 3), or (c) the saccade was hypometric (less than two-thirds of the correct amplitude; 0.2% of trials in Experiment 1, 2.3% in Experiment 2, and 0.6% in Experiment 3). In total, 5.8% of trials were excluded in Experiment 1, 7.2% in Experiment 2%, and 12.5% in Experiment 3.

### Saccade identification

Potential saccades were automatically identified offline using velocity criterion of ≥70°/s. When a potential saccade was identified the algorithm backtracked by five samples and recorded this value. The exact start of the saccade was then found by looking for the first velocity above this smaller pre-start threshold. The raw signal was unfiltered, and the detection algorithm was visually verified for every trial.

## Experiment 1

### Design

Within each block there were four trial types (a) valid trials, where the target appeared at the cued location; (b) invalid trials, where the target appeared contralateral to the cue; (c) Centre cue trials, where the fixation point was cued and the target appeared at one of the two peripheral locations; and (d) Target Absent trials, where the cue appeared but there was no target.

The cue was the removal of one of the two placeholders for 100 ms. In Manual Response blocks, participants were instructed to maintain fixation and to indicate target presence as quickly as possible by pressing the upper button on the response box and the target absence by pressing the lower button (Target Absent trials). Fixation was monitored by recording eye-movements. In saccade response blocks, participants were instructed to make a saccade as quickly and as accurately as possible towards the target or to withhold their response in target absent trials. Each participant completed one block of 20 practice trials and four blocks of 90 experimental trials (two manual responses and two saccade responses). Each block of trials contained 20 valid trials, 20 invalid trials, 20 Centre Cue trials, and 30 Target Absent trials (10 following a left cue, 10 following a right cue, and 10 following a centre cue). Overall, there were 22.22% valid trials, 22.22% invalid trials, 22.22% Neutral trials, and 33.33% Catch trials.

### Results

#### RT

We analysed the RT data from correct responses (84% of trials) with a 2 x 2 x 3 repeated measures analysis of variance (RM ANOVA) with factors of Stimulus Eccentricity (8° and 10°), Response Type (Manual or Oculomotor), and Validity (valid, invalid, and central cue). There was no main effect of Stimulus Eccentricity (*F* = .203), and no interactions between Eccentricity and any of the other factors (all *F*s < 1), so we collapsed across Stimulus Eccentricity for the remaining analyses.

**Figure 2.**
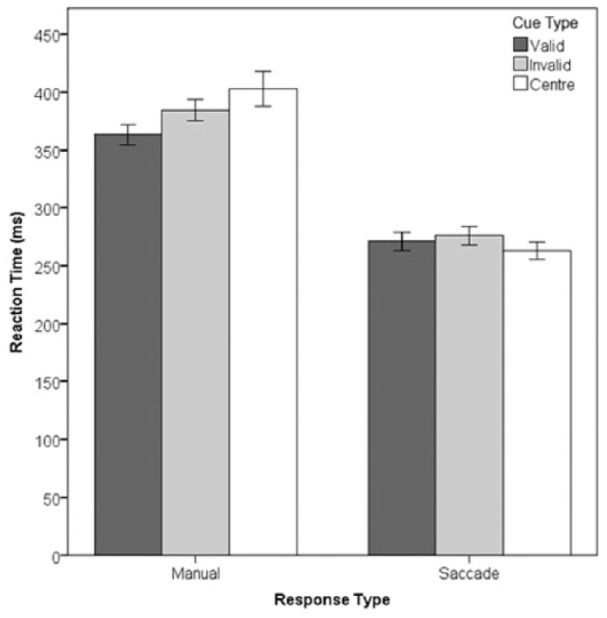
Response type x cue validity interaction. Error bars show within-subject 95% confidence intervals (Cousineau, 2005).

RT data from correct responses are shown in Figure 2. Inspection of Figure 2 suggests the presence of a cueing effect in the manual RT data but not the saccadic RT data. To test this potential interaction effect the median, (S)RT was calculated for all correct responses for each individual. The RTs were then subjected to a 2 × 3 ANOVA with within-subjects factors of Response Type (Manual or Oculomotor) and validity (valid, invalid and central cue). The ANOVA revealed a two-way interaction between Response Type and validity, *F*(2, 32) = 13.37, *p* < .05, $\eta_{p}^{2}=.45$.

The interaction was explored using ANOVAs with a single factor of validity conducted at each level of Response Type. For Manual responses, there was a main effect of validity, *F*(2, 32) = 9.02, *p* < .01, $\eta_{p}^{2}=.36$. Bonferroni corrected paired samples *t*-tests show that the main effect was driven by significant facilitation of RTs on valid trials compared with invalid trials, valid: 368 ms; invalid: 389 ms; *t*(16) = 4.91, *p* < .016, and valid trials compared with Centre trials, valid: 368 ms; Centre: 407 ms; *t*(16) = 3.62, *p* < .016. RTs on invalid trials were also faster than those on Centre trials, but this effect was not significant, valid: 389 ms; Centre: 407 ms; *t*(16) = 1.65, *p* = .12. In contrast, there was no effect of validity in the Saccadic response condition, valid: 276, invalid 278, Centre 264 ms, *F*(2, 32) = 2.36, *p* = .11, $\eta_{p}^{2}=.13$.

#### False alarms

We examined the frequency of erroneous eye-movements on trials in which a cue but no target was presented (Target Absent trials). Overall, the number of erroneous saccades was very low in the Manual Response condition (<1% of trials), so the data are not further described. In contrast, in the Saccade Response condition, participants failed to withhold any saccadic eye-movement on 16% of trials. Table 1 shows the raw frequency of erroneous saccades directed to the left and right in the different cue conditions summed across subjects. The table indicates that erroneous saccades were more common following peripheral cues, and that they were more likely to be directed towards the cued location than the uncued location, χ^2^(2, *n* = 17) = 69, *p* < .05. However, it should be noted that these frequencies are summed across all participants so some of the values may not be truly independent. As a consequence, the results of this test should be interpreted with caution.

**Table 1.** Direction of saccadic errors in the “no target” condition (% of total errors).

	Peripheral left cue (%)	Peripheral right cue (%)	Centre cue (%)
Left saccade	71	15	14
Right saccade	10.7	76	13.3

#### Accuracy

Participants performed the tasks with a high degree of accuracy (97% and 93.7% correct responses on target-present trials in the Manual and Saccadic response conditions, respectively); so, we do not report further analysis of these data.

### Discussion

This study tested the hypothesis that transient offset cues would summon attention without triggering activation of a saccade plan. Consistent with this hypothesis, valid cues produced significant RT facilitation for manual responses but not saccadic response. On first inspection, these data appear to show that attention was oriented to the cued location but that no saccade plan was activated. However, there are several reasons to be cautious about accepting this interpretation. First, we also observed an increased false alarm rate when cues appeared in the periphery in the Saccadic response condition but not the Manual response condition. The fact that saccadic errors were more likely in the peripheral cue condition, and that these errors were systematically biased towards the cued location might be taken as evidence that there was some cue-related oculomotor activation. Second, the proportion of catch trials was relatively high (30%). This is potentially problematic as the high proportion of catch trials meant the likelihood of participants being required to make a saccade to a cued location is relatively low, and Belopolsky and Theeuwes (2009) have argued that oculomotor priming effects are reduced when a saccadic target is unlikely to appear at a cued location.

Experiment 1 failed to show any effect of transient offset on saccadic RT, which might be due to the numerous false alarm response type and the proportion of catch trials. To address these issues, we conducted a second experiment in which we used a saccadic localisation task to assess oculomotor programming and two different measures of covert attention—a manual detection task and a manual discrimination task. The detection task allowed us to directly compare the results of Experiments 1 and 2. However, having a target on every trial introduced the possibility that participants would strategically prepare their response at the start of the trial, rather than wait until target presentation. This strategy could mask any cueing effects. A discrimination task controls for this probability, as the participant cannot pre-prepare a response. If the failure to observe oculomotor priming by offset cues was due to the presence of catch trials, removing catch trials should elicit oculomotor priming in the saccade task and attentional facilitation in the manual detection and discrimination tasks.

## Experiment 2

### Design

Within each block, there were three trial types, such as (a) valid trials, where the target appeared at the cued location; (b) invalid trials, where the target appeared contralateral to the cue; and (c) Centre cue trials, where the fixation point was cued and the target appeared at one of the two peripheral locations. The target appeared at the cued location on one-third of trials. The peripheral cue was the disappearance and the re-appearance of one of the two peripheral placeholders. In the Manual Detection task, participants were instructed to maintain fixation and press a button on the response box as quickly as possible when the target appeared. In the Manual Discrimination task, the response box was aligned, so the buttons lay along the sagittal midline. Participants pressed the upper button for a vertical bar and the lower button for a horizontal bar. In both, these tasks fixation was monitored by recording eye-movements. In the Saccadic Localisation task, participants were instructed to look as quickly as possible at the target. Each participant completed one block of 20 practice trials and six blocks of 60 experimental trials (two Manual Detection, two Manual Discrimination, and two Saccade Localisation). Each block of trials contained 20 valid trials, 20 invalid trials, and 20 central cue trials.

### Results

**Figure 3.**
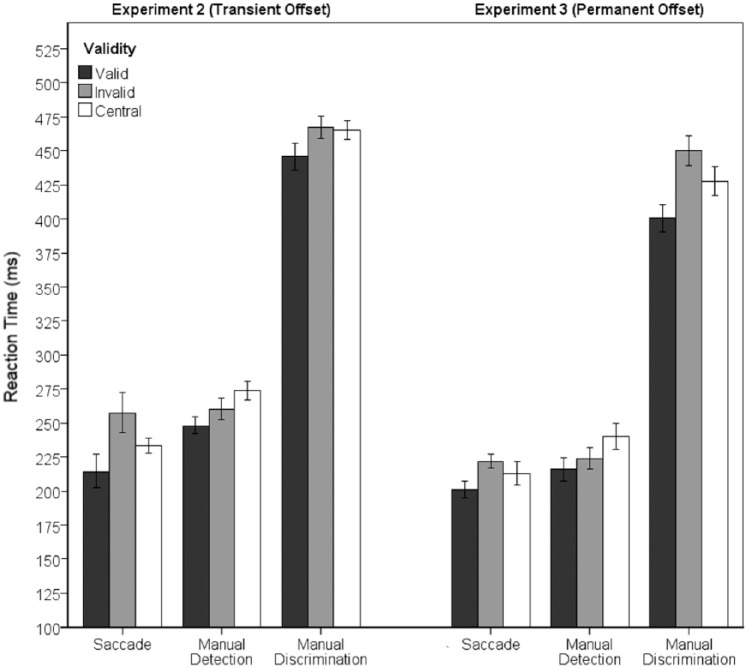
Response type x cue validity interaction in Experiment 2 (left) and Experiment 3 (right). Error bars show within-subject 95% confidence intervals (Cousineau, 2005).

Inspection of Figure 3 suggests that RTs were faster in the valid cue condition than the invalid cue condition in all of the tasks. Unlike Experiment 1, there is clear evidence of facilitation of SRTs. However, while valid trials appear to facilitate RTs for all response types, there appear to be differences in the costs associated with invalid cues. To test this more formally, the median (S)RT were subjected to a 3 × 3 ANOVA with within-subjects factors of Response Type (Saccade, Manual Detection, and Manual Discrimination) and validity (valid, invalid, and central cue). Where the assumption of sphericity was violated, we have reported Geisser–Greenhouse corrected values. The ANOVA revealed a two-way interaction between Response Type and validity, *F*(2, 17.7) = 3.69., *p* < .05, $\eta_{p}^{2}=.29$. One-way ANOVA at each level of Response Type revealed a significant validity effect in all three response types, Saccade: *F*(1.2, 11.6) = 12.03, *p* < .05; Manual Detection: *F*(2, 18) = 11.1, *p* < .05; Manual Discrimination: *F*(2, 18) = 4.07, *p* < .05. However, the pattern of costs/benefits of cueing differed across response types. Specifically, in the Saccadic Localisation task, there were significant RT benefit in the valid condition cues compared with the invalid condition, *t*(9) = 3.67, *p* < .017, and Central condition, *t*(9) = 3.34, *p* < .017, and significant RT costs in the invalid condition compared with the Central condition, *t*(9) = 3.06, *p* < .017. In contrast, in the Manual detection task, the RT facilitation for the valid condition compared with the invalid condition was much less robust, 248 ms versus 260 ms, *t*(9) = 2.16, *p* = .059, and both were faster than the Central cue condition, *t*(9) = 5.63, *p* < .01; *t*(9) = 2.33, *p* = .052, respectively. In the Manual Discrimination task, there was a significant RT benefit in the valid condition compared with the invalid condition, *t*(9) = 2.92, *p* = .017, and the Central condition, *t*(9) = 2.97, *p* = .016, but no cost for invalid condition compared with Centre condition, *t*(9) = .41, *p* = .69. These data are illustrated in Figure 3.

As with Experiment 1, participants performed the tasks with a high degree of accuracy (mean error rate was <4%), so we did not conduct further analysis of these data.

### Discussion

This experiment tested the hypothesis that the failure to observe a cueing effect in the saccadic response condition of Experiment 1 was due to the presence of catch trials, rather than a failure of the cue to trigger saccade programming per se. Consistent with this explanation, removing the catch trials in Experiment 2 led to a reliable facilitation of saccadic RT in the valid condition and a reliable cost in the invalid condition. However, removing catch trials had a different effect on the Manual Detection task, such that the RT facilitation for the valid condition compared with the invalid condition was much reduced. On first inspection, this might suggest that the peripheral cue was less effective at summoning attention. However, given that there were significant cueing effects in the Discrimination task, a more plausible explanation is that the attentional effects of a valid cue in the detection task were masked by the anticipatory effect of knowing that a target would appear on every trial. The fact that RTs were ~100 ms faster in Experiment 2 than Experiment 1 is consistent with this interpretation. Taken together with the false alarm data from Experiment 1, these results suggest that transient offsets elicit both oculomotor preparation and exogenous covert orienting, consistent with the idea that covert exogenous attentional facilitation is tightly coupled with activation of the eye-movement system (Smith & Schenk, 2012).

One potentially important difference between the oculomotor and manual tasks is that the oculomotor task required localisation, whereas the manual tasks do not. It seems likely that using a manual localisation task would have produced results more similar to that saccadic localisation task. However, it is necessary to be cautious when interpreting the results of manual localisation tasks in terms of attentional processing because they confound the validity of a cue with stimulus-response compatibility effects. As a consequence, it is impossible to know whether changes in RT at the cued location are due to enhanced attentional processing, a stimulus-response compatibility effect or some combination of the two.

The results of this experiment suggest that transient offsets elicit both attentional and oculomotor facilitation. However, a transient offset necessarily involves the re-appearance of the cue after it has vanished. Given that object appearance is highly salient, one might argue that using a transient offset does not provide a strong test of the idea that offsets elicit attentional capture but not oculomotor priming. To address this issue we conducted third experiment in which attention was summoned by the permanent removal of the placeholder.

## Experiment 3

### Method

#### Procedure

As is Experiment 2, except that the cue was the permanent offset of one of the peripheral placeholders.

### Results and discussion

The median (S)RT was calculated for all correct responses for each individual. The RTs were then subjected to a 3 × 3 ANOVA with within-subjects factors of Response Type (Oculomotor, Manual Detection, and Manual Discrimination) and validity (valid, invalid, and central cue). Where the assumption of sphericity was violated we have reported the Geisser–Greenhouse corrected values. The ANOVA revealed a two-way interaction between Response Type and validity, *F*(4, 36) = 5.7, *p* < .05, $\eta_{p}^{2}=.39$. One-way ANOVA at each level of response type revealed a significant validity effect in all three response types, Saccade: *F*(2, 18) = 8.02, *p* < .05, $\eta_{p}^{2}=.47$; Manual Detection *F*(2, 18) = 6.8, *p* < .05, $\eta_{p}^{2}=.43$; Manual Discrimination *F*(2, 18) = 18.82, *p* < .05, $\eta_{p}^{2}=.68$. However, the pattern of costs/benefits of cueing differed in the three response types. Specifically, the Saccadic Localisation condition showed significant RT benefits for the valid condition compared with the invalid condition, *t*(9) = 6.08, *p* < .016 but not the Central condition, *t*(9) = 1.86, *p* = .096, and the difference between the invalid Condition and Central condition was not significant, *t*(9) = 1.66, *p* = .13. In contrast, the Manual detection task showed no significant RT facilitation for valid trials compared with invalid trials, *t*(9) = 1.35, *p* = .21, although the valid condition was significantly faster than the Central condition, *t*(9) = 3.3, *p* < .016. The difference between invalid Condition and Central condition was not significant after applying a Bonferroni correction, *t*(9) = 2.41, *p* = .04. Unlike the detection task, in the Manual Discrimination task, there was a significant RT benefit for that valid condition compared with the invalid condition, *t*(9) = 6.23, *p* < .016, and the Central condition, *t*(9) = 3.49, *p* < .016. The difference between invalid Condition and Central condition was not significant after applying the Bonferroni correction, *t*(9) = 2.64, *p* = .027. These interactions are illustrated on Figure 3, right panel. To summarise, valid cues produced robust facilitation in the Saccadic Localisation and Manual Discrimination tasks, and much weak facilitatory effects in the Manual Detection task.

We also conducted an exploratory analysis that directly compared the results of Experiments 2 and 3. Mixed model RM ANOVA with within-participants factors of Response Type (Saccade, Manual Detection, and Manual Discrimination) and validity (valid, Central, and invalid), and a between-subjects factor of Cue Type (Transient and Permanent) produced a Response type x validity interaction, *F*(4, 72) =, *p* < .05, $\eta_{p}^{2}=.15$, and a three-way interaction (*F* = 3.14, *p* < .05, $\eta_{p}^{2}=.15$). The three-way interaction was analysed with 3 (validity) x 2 (Cue Type) ANOVAs at each level of response type. For Saccadic and Manual Detection Responses, there was a main effect of validity, *F*(2, 36) = 19.5, *p* < .05, $\eta_{p}^{2}=.52$; *F*(2, 36) = 16, *p* < .05, *ήp^2^* = .49, respectively, but no effect of Cue Type and no interaction. However, in the Manual Discrimination task, there was a main effect of validity, *F*(2, 36) = 24.5, *p* < .05, $\eta_{p}^{2}=.58$, and a significant validity x Cue Type interaction, *F*(2, 36) = 4.03, *p* < .05, $\eta_{p}^{2}=.18$. This interaction appears to be caused by a significant increase in both the benefits of a valid cue and the costs of an invalid cue in Experiment 3, compared with Experiment 2 (see Figure 3).

## General discussion

In three different experiments, we have shown that peripheral offsets reliably elicit both exogenous covert attention and oculomotor priming. However, the effects were very sensitive to the task context. Specifically, when participants made a saccadic response, the presence of catch trials prolonged saccadic RTs and eliminated the saccadic RT advantage in the valid condition (Experiment 1). Removing the catch trials revealed a significant validity effect in the Saccadic localisation task but greatly reduced the magnitude of the cueing effect in the Manual detection task, probably because participants could begin planning their response as soon as the trial began (Experiments 2 and 3). Consistent with this account, we observed large and robust validity effects for the harder, discrimination task in which the participants could not preprogram their response.

The finding that the presence of catch trials can make it hard to observe facilitation of saccadic RTs by non-predictive, peripheral cues has important implications for the interpretation of a series of studies that use a dual-task method to argue against a coupling between attention and eye-movements (e.g., Hunt & Kingstone, 2003; Klein, 1980; Klein & Pontefract, 1994; MacLean et al., 2015). In these tasks, participants must perform a discrimination task following a predictive peripheral cue. However, on 10%-20% of trials the discrimination target is replaced with a saccade target that participants must fixate as quickly as possible. Klein and colleagues have repeatedly shown that the latency of the saccades towards the attended and unattended location is the same. They argue that the absence of faster saccadic RTs to the attended location means that attention can be deployed without a concurrent saccade plan and conclude that premotor theory (they actually use the term Oculomotor Readiness Hypothesis) is false. However, these experiments contain up to 90% of “no-go” trials, much higher than the 33% we used in Experiment 1. Given our finding that high proportions of catch trials masks oculomotor priming effects in RT data, it may be more appropriate to interpret the null results of Klein and colleagues as “absence of evidence” of oculomotor priming rather than “evidence of absence” of oculomotor priming.

An alternative explanation is that the coupling between covert attention and oculomotor programming depends on the probability that a saccade will be directed to the cued location. In an elegant study, Belopolsky and Theeuwes (2009) observed that when the probability of making a saccade to an attended location was low, covert attentional orienting was preserved but oculomotor priming abolished. They proposed that, consistent with premotor theory, an endogenous shift of attention required activation of a saccade plan. However, they argued that this plan could be rapidly suppressed in cases where the saccade target was likely to be spatial separate from the attended location. In this view, the apparent decoupling between oculomotor programming and exogenous attention observed in our Experiment 1 occurred because the saccade target appeared at the cued location on only 22% of trials, so participants could rapidly suppress cue-induced saccade programming to be ready to make a saccade to the correct location. The saccadic errors on “Catch” trials may have occurred when the suppression of the saccade programme was slow or incomplete. Notably, as with Belopolsky and Theeuwes (2009), the coupling between oculomotor programming and covert attention was restored when the probability of a saccade being directed to the location of a peripheral cue was increased to 50% in Experiment 2. Our data, therefore, complement the findings of Belopolsky and Theeuwes (2009, 2012) by suggesting that dissociation between oculomotor programming and maintenance of endogenous covert attention also pertains to exogenous covert attention.

Why is it that offsets can produce oculomotor priming in the peripheral cueing task but not in the remote distractor task (Hermens & Walker, 2010)? One possibility is that oculomotor priming partly depends on the task context. More specifically, Cole and Kuhn (2010) argued that offsets only capture attention when they are the sole visual transient in the display, or the participant has engaged an attentional set for offsets. Given that offset cues are known to generate relatively small antisaccade costs (Pratt & Trottier, 2005) which suggests they elicit weak activation of the eye-movement system, it may be that the presence or absence of other visual transients in the display is of critical importance for observing oculomotor capture by offsets. In our cueing tasks, the offset was the only visual transient, so even relatively weak activation of the oculomotor system may be sufficient to permit oculomotor capture by the offset. In contrast, in the RDE experiments using offsets, the offset of the distractor is typically accompanied by the onset of a target item. In this case, the target onset signal would be much stronger than the distractor-offset signal, leading to a greatly attenuated RDE. A second possibility is that an offset event is not temporally processed by the oculomotor system the same way as an onset event. During an offset, the system needs to disengage from the spatial location previously activated. One can speculate that this process might affect the timing of target selection. Indeed, Bompas and Sumner (2009) have shown that varying the contrast of a remote distractor systematically alters the stimulus-onset asynchrony (SOA) at which the RDE effect is maximal, and Born and Kerzel (2011) observed that saccade latency is shortened when a target has a higher contrast than a distractor. Given that the optimal SOA for observing the RDE is modulated by the relative contrast of target and distractors and that previous studies of offsets typically use a single, 0 ms gap between target and distractor, it is possible that an RDE to offset distractors might be observed if multiple target-distractor gaps were tested.

To summarise, this study examined whether offset cues could trigger exogenous orienting without engaging saccade programming. The results of Experiments 2 and 3 clearly show that offsets elicit both attentional and oculomotor priming, consistent with the idea that exogenous orienting of attention is tightly coupled to eye-movements. It is argued that studies using the RDM do not observe effects of offsets on saccadic RT because they contain multiple, simultaneous visual transients and the weak activation triggered by the offset of a distractor cannot competed with the strong activation triggered by an onset. In contrast, the Posner cueing task has sequential visual transients. In the absence of competition from other visual transients, even the relatively weak oculomotor activation associated with offsets is sufficient to elicit oculomotor priming and attentional facilitation. We conclude that covert, exogenous orienting is tightly coupled to oculomotor activation, and that previous evidence of dissociations between the two, for example, (Maclean et al., 2015) can be explained by the inclusion of a high proportion of catch trials.

## References

[bibr1-1747021818759468] Awh E. Armstrong K. M. Moore T. (2006). Visual and oculomotor selection: Links, causes and implications for spatial attention. Trends in Cognitive Sciences, 10, 124–130. 16469523 10.1016/j.tics.2006.01.001

[bibr2-1747021818759468] Ball K. Pearson D. G. Smith D. T. (2013). Oculomotor involvement in spatial working memory is task-specific. Cognition, 129, 439–446. 24001480 10.1016/j.cognition.2013.08.006

[bibr3-1747021818759468] Belopolsky A. V. Theeuwes J. (2009). When are attention and saccade preparation dissociated? Psychological Science, 20, 1340–1347. 19788530 10.1111/j.1467-9280.2009.02445.x

[bibr4-1747021818759468] Belopolsky A. V. Theeuwes J. (2012). Updating the premotor theory: The allocation of attention is not always accompanied by saccade preparation. Journal of Experimental Psychology: Human Perception and Performance, 38, 902–914. 22686694 10.1037/a0028662

[bibr5-1747021818759468] Bompas A. Sumner P. (2009). Temporal dynamics of saccadic distraction. Journal of Vision, 9, 17. 10.1167/9.9.17 19761350

[bibr6-1747021818759468] Boot W. R. Kramer A. F. Peterson M. S. (2005). Oculomotor consequences of abrupt object onsets and offsets: Onsets dominate oculomotor capture. Perception & Psychophysics, 67, 910–928. 16334062 10.3758/bf03193543

[bibr7-1747021818759468] Born S. Kerzel D. (2011). Effects of stimulus contrast and temporal delays in saccadic distraction. Vision Research, 51(10), 1163–1172. doi: 21414338 10.1016/j.visres.2011.03.007

[bibr8-1747021818759468] Born S. Mottet I. Kerzel D. (2014). Presaccadic perceptual facilitation effects depend on saccade execution: Evidence from the stop-signal paradigm. Journal of Vision, 14(3), 7. 10.1167/14.3.7 24599945

[bibr9-1747021818759468] Brockmole J. R. Henderson J. M. (2005). Prioritization of new objects in real-world scenes: Evidence from eye movements. Journal of Experimental Psychology-Human Perception and Performance, 31(5), 857–868. doi:. 16262483 10.1037/0096-1523.31.5.857

[bibr10-1747021818759468] Cole G. G. Kuhn G. (2010). Attentional capture by object appearance and disappearance. The Quarterly Journal of Experimental Psychology, 63, 147–159. 19396733 10.1080/17470210902853522

[bibr11-1747021818759468] Cousineau D. (2005). Confidence intervals in within-subject designs: A simpler solution to Loftus and Masson’s method. Tutorial in Quantitative Methods for Psychology, 1(1), 4–45.

[bibr12-1747021818759468] Craighero L. Carta A. Fadiga L. (2001). Peripheral oculomotor palsy affects orienting of visuospatial attention. Neuroreport, 12, 3283–3286. 11711871 10.1097/00001756-200110290-00027

[bibr13-1747021818759468] Dunne S. Ellison A. Smith D. T. (2015). Rewards modulate saccade latency but not exogenous spatial attention but not exogenous spatial attention. Frontiers in Psychology, 6, 1080. 10.3389/fpsyg.2015.01080 PMC4516812 26284004

[bibr14-1747021818759468] Gabay S. Henik A. Gradstein L. (2010). Ocular motor ability and covert attention in patients with Duane Retraction Syndrome. Neuropsychologia, 48, 3102–3109. 20600187 10.1016/j.neuropsychologia.2010.06.022

[bibr15-1747021818759468] Hermens F. Walker R. (2010). The influence of onsets and offsets on saccade programming. I-perception, 1, 83–94. 23397028 10.1068/i0392 PMC3563056

[bibr16-1747021818759468] Hopfinger J. B. Mangun G. R. (1998). Reflexive attention modulates processing of visual stimuli in human extrastriate cortex. Psychological Science, 9, 441–447. 26321798 10.1111/1467-9280.00083 PMC4552358

[bibr17-1747021818759468] Hopfinger J. B. Mangun G. R. (2001). Tracking the influence of reflexive attention on sensory and cognitive processing. Cognitive, Affective, & Behavioral Neuroscience, 1, 56–65. 10.3758/cabn.1.1.56 12467103

[bibr18-1747021818759468] Hunt A. R. Kingstone A. (2003). Covert and overt voluntary attention: Linked or independent? Cognitive Brain Research, 18, 102–105. 14659502 10.1016/j.cogbrainres.2003.08.006

[bibr19-1747021818759468] Klein R. M. (1980). Does oculomotor readiness mediate cognitive control of visual attention? In Nickerson R. (Ed.), Attention and performance (Vol. IX, pp. 259–276). Hillsdale, MI: Lawrence Erlbaum.

[bibr20-1747021818759468] Klein R. M. Pontefract A. (1994). Does oculomotor readiness mediate cognitive control of visual-attention? Revisited. In Umiltà C. Moscovitch M. (Eds.), Attention and Performance Xv (Vol. 15, pp. 333–350). Cambridge: The MIT Press.

[bibr21-1747021818759468] Ludwig C. J. H. Ranson A. Gilchrist I. D. (2008). Oculomotor capture by transient events: A comparison of abrupt onsets, offsets, motion, and flicker. Journal of Vision, 8(14), 1–16. 10.1167/8.14.11 19146312

[bibr22-1747021818759468] MacLean G. H. Klein R. M. Hilchey M. D. (2015). Does oculomotor readiness mediate exogenous capture of visual attention? Journal of Experimental Psychology: Human Perception and Performance, 41, 1260–1270. 26076176 10.1037/xhp0000064

[bibr23-1747021818759468] Morgan E. J. Ball K. Smith D. T. (2014). The role of the oculomotor system in covert social attention. Attention Perception & Psychophysics, 76, 1265–1270. 10.3758/s13414-014-0716-1 24944104

[bibr24-1747021818759468] Pearson D. G. Ball K. Smith D. T. (2014). Oculomotor preparation as a rehearsal mechanism in spatial working memory. Cognition, 132, 416–428. 24908341 10.1016/j.cognition.2014.05.006

[bibr25-1747021818759468] Posner M. I. (1980). Orienting of attention. Quarterly Journal of Experimental Psychology, 32(FEB), 3–25. 7367577 10.1080/00335558008248231

[bibr26-1747021818759468] Pratt J. McAuliffe J. (2001). The effects of onsets and offsets on visual attention. Psychological Research/Psychologische Forschung, 65, 185–191. 11571913 10.1007/s004260100058

[bibr27-1747021818759468] Pratt J. Trottier L. (2005). Pro-saccades and anti-saccades to onset and offset targets. Vision Research, 45, 765–774. 15639503 10.1016/j.visres.2004.05.019

[bibr28-1747021818759468] Rafal R. D. Posner M. I. Friedman J. H. Inhoff A. W. Bernstein E. (1988). Orienting of visual-attention in progressive supranuclear palsy. Brain, 111(Pt 2), 267–280. 3378136 10.1093/brain/111.2.267

[bibr29-1747021818759468] Riggio L. Bello A. Umilta C. (1998). Inhibitory and facilitatory effects of cue onset and offset. Psychological Research/Psychologische Forschung, 61, 107–118. 9689907 10.1007/s004260050017

[bibr30-1747021818759468] Rizzolatti G. Riggio L. Sheliga B. M. (1994). Space and selective attention. In Umiltà C. Moscovitch M. (Eds.), Attention and Performance Xv (Vol. 15, pp. 231–265). Cambridge: The MIT Press.

[bibr31-1747021818759468] Sheliga B. M. Riggio L. Rizzolatti G. (1994). Orienting of attention and eye-movements. Experimental Brain Research, 98, 507–522. 8056071 10.1007/BF00233988

[bibr32-1747021818759468] Smith D. T. Ball K. Ellison A. (2014). Covert visual search within and beyond the effective oculomotor range. Vision Research, 95, 11–17. 24333751 10.1016/j.visres.2013.12.003

[bibr33-1747021818759468] Smith D. T. Ball K. Ellison A. Schenk T. (2010). Deficits of reflexive attention induced by abduction of the eye. Neuropsychologia, 48, 1269–1276. 20036265 10.1016/j.neuropsychologia.2009.12.028

[bibr34-1747021818759468] Smith D. T. Rorden C. Jackson S. R. (2004). Exogenous orienting of attention depends upon the ability to execute eye movements. Current Biology, 14, 792–795. 15120071 10.1016/j.cub.2004.04.035

[bibr35-1747021818759468] Smith D. T. Rorden C. Schenk T. (2012). Saccade preparation is required for exogenous attention but not endogenous attention or IOR. Journal of Experimental Psychology: Human Perception and Performance, 36, 1438–1447. 10.1037/a0027794 22428677

[bibr36-1747021818759468] Smith D. T. Schenk T. (2012). The premotor theory of attention: Time to move on? Neuropsychologia, 50, 1104–1114. 22306518 10.1016/j.neuropsychologia.2012.01.025

[bibr37-1747021818759468] Sumner P. Adamjee T. Mollon J. D. (2002). Signals invisible to the collicular and magnocellular pathways can capture visual attention. Current Biology, 12, 1312–1316. 12176359 10.1016/s0960-9822(02)01020-5

[bibr38-1747021818759468] Todd J. T. Vangelder P. (1979). Implications of a transient-sustained dichotomy for the measurement of human-performance. Journal of Experimental Psychology: Human Perception and Performance, 5, 625–638. 528963 10.1037//0096-1523.5.4.625

